# TESTING THE ROLE OF MITOCHONDRIAL DYSFUNCTION IN AGE-RELATED HYPERTENSIVE AORTIC DISEASE

**DOI:** 10.1093/geroni/igae098.3303

**Published:** 2024-12-31

**Authors:** Arjune Dhanekula, Scott DeRoo, Christopher Burke, Billie Hwang, Michael Mulligan, Jay Pal, David Marcinek

**Affiliations:** University of Washington, Seattle, Washington, United States; University of Washington, Seattle, Washington, United States; University of Washington, Seattle, Washington, United States; University of Washington, Seattle, Washington, United States; University of Washington, Seattle, Washington, United States; University of Washington, Seattle, Washington, United States; University of Washington, Seattle, Washington, United States

## Abstract

Aging is a risk factor for hypertension, aortic aneurysm, and dissection. However, the age-related changes in aortic biology underlying this risk are poorly understood. Mitochondrial dysfunction is an important contributor of both aging and cardiovascular pathology. However, most models of aortic disease are in young animals, lacking the context of age-related changes. To test whether mitochondrial changes drive aortic aneurysm, we developed a unique mouse model replicating age-related hypertensive aortic disease. A pilot study used multiple doses of angiotensin II (AngII) for 4 weeks to test the development of aortic disease in 20 month C57Bl/6 mice (n=8). These data revealed a statistically significant increase in ascending aortic size for AngII doses ⩾1000 ng/kg/min (1.603 +/- 0.054 mm versus 1.433 +/- 0.064 mm, p< 0.05). A cohort of older C57Bl/6 mice (23-24 months, n=22) were then treated with AngII (1500 ng/kg/min) or saline, with no change in aortic size over the 4 week study period. The older cohort also revealed no difference in maximal oxygen consumption rate (OCR) between the AngII and saline groups (30 +/- 3.6 versus 29.4 +/- 0.9 pmol/(sec*mg), p=0.99). However, the 20 month mice in the pilot data had a significantly higher maximal OCR (58 +/- 13.4 pmol/(sec*mg), p< 0.0001). These results demonstrate that angiotensin II administration in older mice results in the development of aortic aneurysm in an age-dependent manner, with an unexpected increase in maximal mitochondrial respiratory capacity associated with disease development. These results highlight the importance of testing age-related disease in the context of aging biology.

